# Placental Malaria: Hypertension, VEGF, and Prolactin

**DOI:** 10.1371/journal.pmed.0040141

**Published:** 2007-03-27

**Authors:** Roy Douglas Pearson

The findings by Muehlenbachs et al. [1] that placental malaria (PM) is associated with hypertension in first-time mothers aged 18–20 years is significant, and not to be explained at this time of writing. The authors also provide data suggesting that the maternal–fetal conflict, during chronic PM and hypertension in first-time mothers, involves the VEGF pathway.

Previously [2–5], I have posited that prolactin might have a role in PM and these new findings might provide further indirect evidence for such a role. It should be remembered that there is an extensive and decades-old literature (see Horrobin's chapter 23 in [6]) on the role of prolactin in hypertension; and more specifically, the relationship between prolactin and pregnancy-related hypertension [7,8].

Regarding the VEGF pathway, Malaguarnera et al. [9] have recently shown that prolactin induces VEGF production in human macrophages. It is conceivable that hyperprolactinemia (pituitary and/or placental) could up-regulate placental macrophage production of VEGF.

Space does not permit a discussion of the well known fact of increased pregnancy-related prolactin in first-time mothers, but this has been noted elsewhere [2] concerning maternal malaria.

Although there has been controversy of late [2,10], regarding my “prolactin hypothesis” in maternal malaria, it is time definitive experiments be conducted to ascertain if prolactin is playing a role in PM, and in other infectious diseases as well.
